# Attitude Estimation Algorithm of Portable Mobile Robot Based on Complementary Filter

**DOI:** 10.3390/mi12111373

**Published:** 2021-11-08

**Authors:** Mei Liu, Yuanli Cai, Lihao Zhang, Yiqun Wang

**Affiliations:** School of Automation Science and Engineering, Xi’an Jiaotong University, Xi’an 710049, China; liumei1997@foxmail.com (M.L.); 13772187823@163.com (L.Z.); 3121154032@stu.xjtu.edu.cn (Y.W.)

**Keywords:** portable mobile robot, quaternion implementation, complementary filter, extended Kalman filtering, attitude estimation

## Abstract

In robot inertial navigation systems, to deal with the problems of drift and noise in the gyroscope and accelerometer and the high computational cost when using extended Kalman filter (EKF) and particle filter (PF), a complementary filtering algorithm is utilized. By combining the Inertial Measurement Unit (IMU) multi-sensor signals, the attitude data are corrected, and the high-precision attitude angles are obtained. In this paper, the quaternion algorithm is used to describe the attitude motion, and the process of attitude estimation is analyzed in detail. Moreover, the models of the sensor and system are given. Ultimately, the attitude angles are estimated by using the quaternion extended Kalman filter, linear complementary filter, and Mahony complementary filter, respectively. The experimental results show that the Mahony complementary filtering algorithm has less computational cost than the extended Kalman filtering algorithm, while the attitude estimation accuracy of these two algorithms is similar, which reveals that Mahony complementary filtering is more suitable for low-cost embedded systems.

## 1. Introduction

A portable mobile robot is a cable-free remote control, semi-autonomous mobile platform system that can assist or even replace workers in completing dangerous tasks. It can be used in anti-terrorism, mines, disaster rescue, field ground pipeline inspections, and security inspections. Attitude estimation is a basic and significant part in robot motion control, whose speed and accuracy will directly affect the stability and reliability of robot’s motion.

The navigation system provides the attitude information of the mobile robot and determines the remote-control direction. The inertial navigation system (INS) is an autonomous navigation system that does not rely on any external information or radiates energy to the outside, which has the characteristics of good concealment and can be used in the air, ground, underwater, and other complex environments. At present, with the help of gyroscopes and accelerometers in the inertial measurement unit, the attitude data of robot are calculated by using the real-time data of the sensor [1,2]. The system establishes the navigation coordinate system by utilizing the output of the gyro, and it calculates the speed and position of the carrier in the navigation system by utilizing the output of the accelerometer [3]. In the research of robot systems, the accuracy of the robot’s attitude estimation will affect the robot’s positioning and navigation. 

In flight and robot control systems, inertial navigation systems are usually composed of Micro-Electro-Mechanical System (MEMS) devices, due to their low cost, small size, easy integration, and low power consumption [4]. In MEMS-based INS, the three-axis gyroscope and accelerometer are used to measure the angular movement and linear movement information, respectively, and the microcomputer calculates the steer, attitude, speed and position based on the measurement information. The gyroscope measures the derivative of the angle, which is the angular velocity, and the angle can be calculated by integrating the angular velocity with time. However, the error of the measured angle accumulates continuously with integration due to the noise, which results from low-frequency interference and drift. At the same time, the accelerometer outputs the current acceleration (including the gravitational acceleration *g*). However, the accelerometer is sensitive to high-frequency signals for its mechanism, which results from high-frequency interference in vibration environments [5]. Thus, it is worthwhile to study how to combine the data from these sensors to filter the measurement noise and system noise of the inertial navigation system and get high-precision attitude data.

It is imperative to handle the measurement noise and system noise of the inertial navigation system during robot attitude estimation. There are three commonly used methods, namely complementary filtering, Kalman filtering, and the gradient descent method. Different results vary from algorithm to algorithm. Yuan Lao-hu et al. [6] introduced adaptive complementary filtering, gradient-descent, and complementary filtering, and they compared the results of the attitude estimation in static and dynamic aspects. D. Choukroun et al. [7] presented a novel Kalman filter (KF) for estimating the attitude quaternion as well as gyro random drifts from vector measurements by employing a special manipulation on the measurement equation. Wang Xiaoxu et al. [5] estimated the attitude of the two-wheeled robot by using Extended Kalman Filter (EKF). Ya-qi et al. [8] and Miaomiao et al. [9] proposed a method to measure the missile attitude based on an extended Kalman filter. He used EKF to linearize the nonlinear model to improve the accuracy and stability of the system. However, EKF needs a large amount of calculation, due to the required Jacobian matrix. What is worse, in a strong nonlinear system, EKF has a large truncation error and is affected by non-Gaussian noise, which may result in divergence [10]. The matching trajectory adopted by Ya-qi et al. was different from the real revolution. In the algorithm of Miaomiao et al., only the first order of the Taylor expansion is carried out, which quickly leads to filter divergence. Liang Song et al. [11] improved the Sage–Husa adaptive filter algorithm based on existing methods to simplify the structure of the algorithm. By estimating the noise covariance with the forgetting factor, the presented algorithm improved the efficiency and accuracy of the attitude estimation. Similarly, Li Gang et al. [12] introduced the improved Sage–Husa adaptive extended Kalman filter. By improving the value of the measured noise, the robustness of the algorithm is improved, and the driving state of the vehicle is accurately estimated. Zhang Xiaojun et al. [13] adopted Particle Filter (PF) to improve the filtering accuracy, which effectively overcomes the strong nonlinearity and filtering divergence problems that EKF may encounter. However, particle filtering is a statistical filtering method based on Monte Carlo simulation. It approximates the nonlinear system by predicting and updating the function sampling set from the system probability density function, so a lot of computations are required, which is not advisable for low-cost robot system attitude calculation [14].

The dynamic response characteristics of the gyroscope are good, but it has drift characteristics, so the long-running drift is severe, and the integrated operation generates cumulative error. On the contrary, the digital compass and accelerometer have no accumulated error in measuring attitude, but the dynamic response is less impressive. In general, they have complementary characteristics in the frequency domain, and complementary filters can be used to fuse the data of these sensors to improve the measurement accuracy and the dynamic performance of the system. Complementary filtering algorithms are widely used in aircraft attitude estimation owing to its small computation, high reliability, and low accuracy requirements for inertial devices [15,16,17].

**Figure 1:**
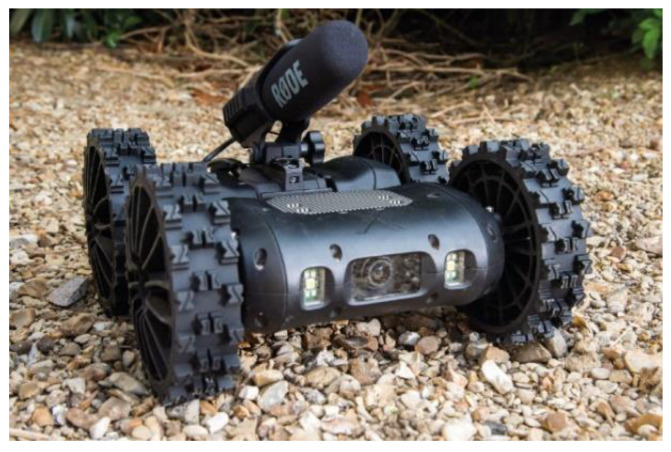
NERVA LG Robot [18].

This paper studies four-wheel drive skid-steering mobile robots (SSMR), whose independent driving wheels can provide large horse power with great obstacle climbing ability, as shown in Figure 1. The NERVA LG Robot in Figure 1 uses four motors to drive four wheels equipped with various sensors and other equipment as a mobile platform, to perform reconnaissance tasks in field conditions [18].

The existing mobile robots filter the measurement and system noise of the inertial navigation system based mostly on Kalman filtering and particle filtering algorithm. Faced with the shortcomings of extended Kalman filter and particle filter applied to robot attitude calculation, this paper adopts the complementary filtering algorithm to compensate and correct the measured attitude data by fusing the data of IMU multi-sensor to calculate the high-precision attitude angles. The novelties and contributions of this paper are listed as follows.

(1) Attitude estimation is a basic and significant part in robot motion control, whose speed and accuracy will directly affect the stability and reliability of the robot’s motion. This paper compares the quaternion extended Kalman filtering, linear complementary filtering, and Mahony complementary filtering algorithms in the field of attitude estimation. The attitude precision and computational cost is discussed.
(2) Portable mobile robots can be used in anti-terrorism, mines, disaster rescue, field ground pipeline inspections, and security inspections. Confronted with the problem of high computational cost when using extended Kalman filter, this paper introduces the Mahony filtering algorithm (widely used in flight attitude estimation) into portable mobile robots and validates that this algorithm is suitable for low-cost embedded systems.

## 2. System Descriptions

### 2.1. Definition of Coordinates and Attitude Description

**Figure 2:**
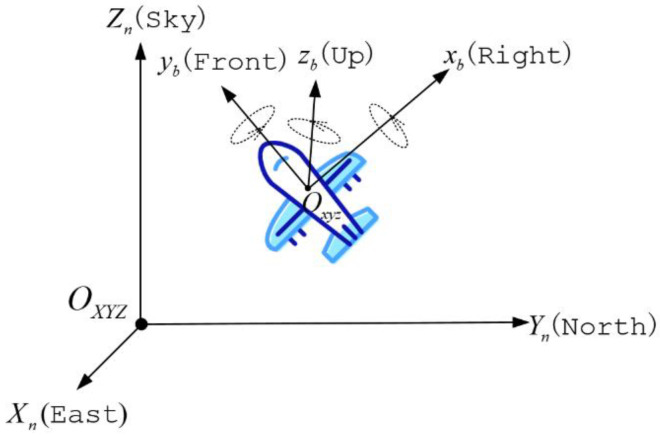
Definition of navigation and carrier coordinate system.

**Figure 3:**
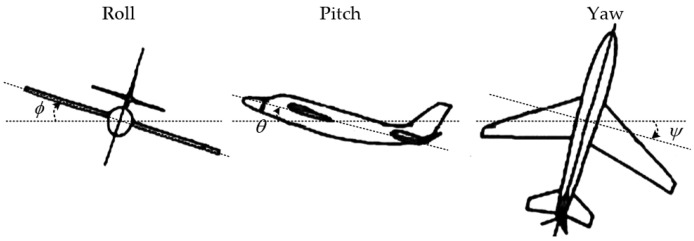
Schematic diagram of body attitude angles (Roll–Pitch–Yaw).

A coordinate system needs to be established before attitude description. The Right-Front-Up coordinate system is selected as the carrier coordinate system (coordinate b), as is shown in Figure 2. $O_{xyz}$ is the carrier coordinate system, where the right direction of the body is the positive direction of the x-axis, the forward direction of the carrier is the positive direction of the y-axis, and the upward direction is the positive direction of the z-axis. The carrier coordinate system is fixedly connected with the body, and the coordinate origin is the center of the body. The mobile robot’s attitude can be expressed by its pitch angle θ, roll angle ϕ, and yaw angle ψ in the Right-Front-Up coordinate system, which rotates around the x-axis, y-axis, and z-axis, respectively. Figure 3 shows the schematic diagram of the body attitude angles.

The position of the robot is determined by the navigation coordinate system (coordinate n), which is an Earth-referenced coordinate system. The East–North–Sky coordinate system is selected as the navigation coordinate system. As is shown in Figure 2, $O_{XYZ}$ is the carrier coordinate system, and we define the positive directions of the x-axis, y-axis, and z-axis as East, North, and Sky.

During the movement of the robot, the carrier coordinate system keeps moving with the robot, while the navigation system remains fixed. Since the IMU is fixed on the robot, the data measured by the IMU are based on the carrier coordinate system. When estimating the attitude, it is necessary to convert the data of the carrier system to the navigation system according to the following equation:

(1) $$\begin{array}{l} \left[\begin{array}{l} X\\ Y\\ Z \end{array}\right]=C_{b}^{n}\left[\begin{array}{c} x\\ y\\ z \end{array}\right] \end{array}$$

where $C_{b}^{n}$ is the attitude transformation matrix, which is expressed as

(2) $$C_{b}^{n}=\left[\begin{array}{ccc} \cos\theta \cos\psi & \sin\theta \sin\phi \cos\psi -\cos\phi \sin\psi & \sin\theta \cos\phi \cos\psi +\sin\phi \sin\psi \\ \cos\theta \sin\psi & \sin\theta \sin\phi \sin\psi +\cos\phi \cos\psi & \sin\theta \cos\phi \sin\psi -\sin\phi \cos\psi \\ -\sin\theta & \sin\phi \cos\theta & \cos\phi \cos\theta \end{array}\right].$$

Equation (2) can be expressed as

(3) $$C_{b}^{n}=\left[\begin{array}{ccc} T_{11} & T_{12} & T_{13}\\ T_{21} & T_{22} & T_{23}\\ T_{31} & T_{32} & T_{33} \end{array}\right].$$

According to Equation (3), the desired attitude angles can be expressed as

(4) $$\{\begin{array}{cc} \phi =\arctan\left(\frac{T_{32}}{T_{33}}\right) & \left(-\pi ,\pi \right)\\ \theta =\arcsin\left(-T_{31}\right) & \left(-\frac{\pi }{2},\frac{\pi }{2}\right)\\ \psi =\arctan\left(\frac{T_{21}}{T_{11}}\right) & \left(-\pi ,\pi \right) \end{array}.$$

However, once the Euler angle method is used, if the pitch angle is near $\pm 90^{\circ }$, it will appear to have singular points [19], and the Euler angle method is stuck in Gimbal Lock problem. Therefore, the quaternion method that converts the data from IMU into a quaternion is introduced to solve this problem.

Quaternion is a hyper complex number. For any quaternion,

(5) $$Q=q_{0}+q_{1}\vec{i}+q_{2}\vec{j}+q_{3}\vec{k}$$

$q_{0},q_{1},q_{2},q_{3}$ are all real numbers, and i,j,k are mutually orthogonal unit vectors. With the quaternion, the attitude transformation matrix from the carrier coordinate system to the navigation coordinate system can be obtained as

(6) $$C_{b}^{n}=\left[\begin{array}{ccc} q_{0}^{2}+q_{1}^{2}-q_{2}^{2}-q_{3}^{2} & 2\left(q_{1}q_{2}-q_{0}q_{3}\right) & 2\left(q_{1}q_{3}+q_{0}q_{2}\right)\\ 2\left(q_{1}q_{2}+q_{0}q_{3}\right) & q_{0}^{2}-q_{1}^{2}+q_{2}^{2}-q_{3}^{2} & 2\left(q_{2}q_{3}-q_{0}q_{1}\right)\\ 2\left(q_{1}q_{3}-q_{0}q_{2}\right) & 2\left(q_{2}q_{3}+q_{0}q_{1}\right) & q_{0}^{2}-q_{1}^{2}-q_{2}^{2}+q_{3}^{2} \end{array}\right].$$

The desired attitude angles can be expressed as

(7) $$\{\begin{array}{cc} \phi =\arctan\frac{2\left(q_{2}q_{3}-q_{0}q_{1}\right)}{q_{0}{}^{2}-q_{1}{}^{2}-q_{2}{}^{2}+q_{3}{}^{2}} & \left(-\pi ,\pi \right)\\ \theta =-\arcsin2\left(q_{1}q_{3}-q_{0}q_{2}\right) & \left(-\frac{\pi }{2},\frac{\pi }{2}\right)\\ \psi =\arctan\frac{2\left(q_{1}q_{2}+q_{0}q_{3}\right)}{q_{0}{}^{2}-q_{1}{}^{2}-q_{2}{}^{2}+q_{3}{}^{2}} & \left(-\pi ,\pi \right) \end{array}.$$

### 2.2. Mathematical Model of Sensors

The measurement error of gyroscopes is composed of random drift and random noise. The mathematical model of gyroscopes can be expressed as

(8) $$\Omega_{y}=\Omega +b+n$$

where Ω_y_ is the measured value of the angular velocity, Ω is the true value of angular velocity, *b* is the time-varying drift, and *n* is the random noise, which can be treated as Gaussian white noise.

The mathematical model of accelerometers can be expressed as

(9) $$a=C_{b}^{n}\left(\dot{v}-g_{n}\right)+b_{a}+n_{a}$$

where ***a*** is the measured value of accelerometers, $C_{b}^{n}$ is the attitude transformation matrix from the carrier system to the navigation system, ***g****_n_* is the gravitational acceleration in navigation system, *b_a_* is the time-varying drift, and $n_{a}$ is the random noise.

The mathematical model of magnetometers can be given by

(10) $$m=C_{b}^{n}m_{I}+B_{m}+n_{b}$$

where ***m****_I_* is the geomagnetic field vector in the navigation coordinate system, *B_m_* is the current magnetic interference, and *n_b_* is the random noise.

## 3. Complementary Filtering Algorithms

In inertial measuring units (IMU), there are two basic methods for calculating the attitude angles. One is to get the attitude angles by integrating the angular velocity from gyroscopes:

(11) $${angle}_{\Omega_{y}}=angle_{0}+\int_{0}^{t}\Omega_{y}dt$$

where *angel*_0_ is the initial angle of the body, Ω_y_ is the measured value of the gyroscopes’ angular velocity.

The other way is to get the attitude angles by orthogonally decomposing the acceleration. The accelerometer outputs the three-axis acceleration in the carrier coordinate system, and the magnetometer outputs the three-axis geomagnetic intensity in the carrier coordinate system. The accelerometer can calculate the pitch and roll angles, and the magnetometer calculates the yaw angle [20]. These two sensors cooperate with each other to solve three attitude angles:

(12) $$\{\begin{array}{ll} \phi =\arctan\frac{a_{y}}{a_{z}} & \left(-\pi ,\pi \right)\\ \theta =\arcsin-\frac{a_{x}}{g} & \left(-\frac{\pi }{2},\frac{\pi }{2}\right)\\ \psi =\arctan\frac{m_{y}^{n}}{m_{x}^{n}} & \left(-\pi ,\pi \right) \end{array}$$

where $a_{b}={\left[\begin{array}{ccc} a_{x} & a_{y} & a_{z} \end{array}\right]}^{T}$ is the output of accelerometers in the carrier coordinate system; $a_{n}={\left[\begin{array}{ccc} 0 & 0 & g \end{array}\right]}^{T}$ is the output of accelerometers in the navigation system when the body is stationary; *g* is the gravitational acceleration; and $m_{n}={\left[\begin{array}{ccc} m_{x}^{n} & m_{y}^{n} & m_{z}^{n} \end{array}\right]}^{T}$ is the magnetic induction intensity of the body in the navigation system.

The accelerometer has better static stability, while it is susceptible to high-frequency signals, which imbues the data with unreliability in vibration environments. On the contrary, the gyroscope has better dynamic stability, while its data are relatively unreliable in a stable environment. Therefore, complementary filtering algorithms have been proposed to combine the date of these sensors to filter external interference and get high reliability and high-precision attitude data [21,22,23,24,25]. By using the complementary relationship between accelerometers and gyroscopes in the frequency domain, this algorithm fuses the body’s attitude data and corrects the drift error of the gyroscope. In other words, the complementary filtering algorithm corrects the integration error of the gyroscope relying on the output stability of the accelerometer. When the body is kept horizontal, accelerometers fail to measure the rotation quantity around the z-axis (yaw angle *ψ*), which is the same as magnetometers. Therefore, the accelerometer and magnetometer are required to make joint efforts to correct the gyroscope.

### 3.1. Linear Complementary Filtering

**Figure 4:**
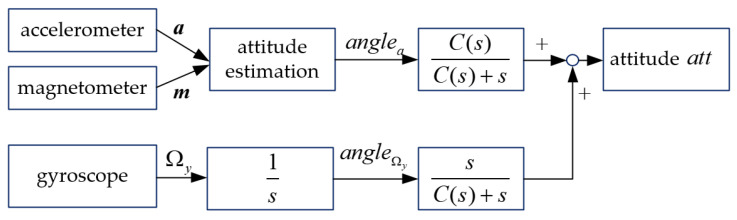
Principle of classic complementary filtering.

The principle of classic linear complementary filtering (CCF) [21] is shown in Figure 4.

In Figure 4, ***a*** is the measured value of accelerometers; ***m*** is the measured value of magnetometers; Ω_y_ is the measured value of the gyroscopes’ angular velocity; *angel_a_* is the attitude angles according to Equation (12); $angle_{\Omega_{y}}$ is the attitude angles according to Equation (11); $att={\left[\begin{array}{ccc} \phi & \theta & \psi \end{array}\right]}^{T}$ is the body’s attitude; 1/s stands for integrator; C(s)/(C(s)+s) is the low-pass filter, and s/(C(s)+s) is the high-pass filter. When *C*(*s*) is a constant, it corresponds to a first-order filter, and when *C(s) = a + b/s*, it corresponds to a second-order filter.

According to Figure 4, the attitude of the body can be obtained as below

(13) $$att=\frac{C(s)}{C(s)+s}angle_{a}(s)+\frac{\Omega_{y}}{C(s)+s}.$$

If implementing the classic linear complementary filtering (CCF) algorithm in terms of a first-order filter, i.e., C(s)=1/τ, then Equation (13) can be written as

(14) $$att=\frac{1/\tau }{1/\tau +s}angle_{a}(s)+\frac{\Omega_{y}}{1/\tau +s}=\frac{1}{\tau s+1}angle_{a}(s)+\frac{\Omega_{y}\tau }{\tau s+1}.$$

Implementing the first-order backward difference to Equation (14) according to:

(15) $$s=\frac{1-z^{-1}}{T_{s}},$$

we can get the discrete time difference form of CCF:

(16) $$att(k)=\frac{\tau }{\tau +T_{s}}\left(att(k-1)+T\Omega_{y}\left(k\right)\right)+\frac{T_{s}}{\tau +T_{s}}angle_{a}(k).$$

where *τ* is the time constant, which can be calculated with Equation (17):

(17) $$f_{c}=\frac{1}{2\pi \tau }$$

where *f_c_* is the cut-off frequency, which can be obtained by analyzing the signal from the sensors in the frequency domain.

### 3.2. Mahony Complementary Filtering

Even though linear complementary filtering (CCF) can eliminate the high-frequency interference of accelerometers as well as the low-frequency interference of gyroscopes by fusing the attitude data, when in high noise, the filtering result is less impressive due to the slow attenuation of the low-pass stopband of CCF. Hence, the Mahony complementary filtering algorithm is proposed, which is a nonlinear complementary filtering algorithm [18].

Define the gravitational acceleration in the navigation coordinate system as **g***_n_*, where $g_{n}={\left[\begin{array}{ccc} 0 & 0 & 1 \end{array}\right]}^{T}$. Subsequently, convert **g***_n_* in the navigation system into **g***_b_* into a carrier coordinate system according to Equation (18).

(18) $$g_{b}=C_{n}^{b}g_{n}={\left(C_{b}^{n}\right)}^{T}g_{n}$$

where $C_{b}^{n}$ is the attitude transformation matrix from the carrier system to the navigation system, as shown in Equation (6). $C_{n}^{b}$ is the transformation matrix from the navigation system to the carrier system. Substitute Equation (6) into Equation (18), and we can get

(19) $$g_{b}=\left[\begin{array}{c} 2\left(q_{1}q_{3}-q_{0}q_{2}\right)\\ 2\left(q_{2}q_{3}+q_{0}q_{1}\right)\\ q_{0}^{2}-q_{1}^{2}-q_{2}^{2}+q_{3}^{2} \end{array}\right].$$

It can be seen from Equation (19) that the theoretical gravitational acceleration in a carrier coordinate system is equal to the last column of $C_{b}^{n}$ in Equation (6). Therefore, the gravitational acceleration can be obtained with the quaternion attitude. In addition, we can also measure the actual gravitational acceleration by utilizing accelerometers. There is a deviation between the theoretical gravitational acceleration and the actual gravitational acceleration, which is largely caused by the angular velocity error generated by the gyroscope signal. Therefore, the error of the gyroscope signal can be compensated in the light of these two gravitational accelerations, and then, a more accurate attitude can be calculated.

Define the output of accelerometers in the carrier coordinate system as *a*. The acceleration after normalization can be obtained as

(20) $$a_{b}=\left[\begin{array}{ccc} \frac{a_{x}}{\left|a\right|} & \frac{a_{y}}{\left|a\right|} & \frac{a_{z}}{\left|a\right|} \end{array}\right].$$

Owing to the deviation between the theoretical gravitational acceleration and the actual gravitational acceleration, the compensation value *e* can be obtained by calculating the cross product of **g***_b_* in Equation (19) and $a_{b}$ in Equation (20). In order to compensate for the gyroscope, each of the correction vectors (yaw and roll-pitch) are fed to a proportional plus integral (PI) feedback controller to be added to the gyro vector to produce a corrected gyro vector [22]. The equation is shown as follows:

(21) $$\delta =K_{p}e+K_{i}\int edt$$

where ***δ*** is the compensation vector of gyroscopes, *K_p_* is the proportional gain, and *K_i_* is the integral gain, respectively.

After the compensation value is added to the output of gyroscopes, the quaternion value can be obtained by using the data of the gyroscope. Ultimately, by converting the quaternion into the Euler angle, the attitude can be estimated.

**Figure 5:**
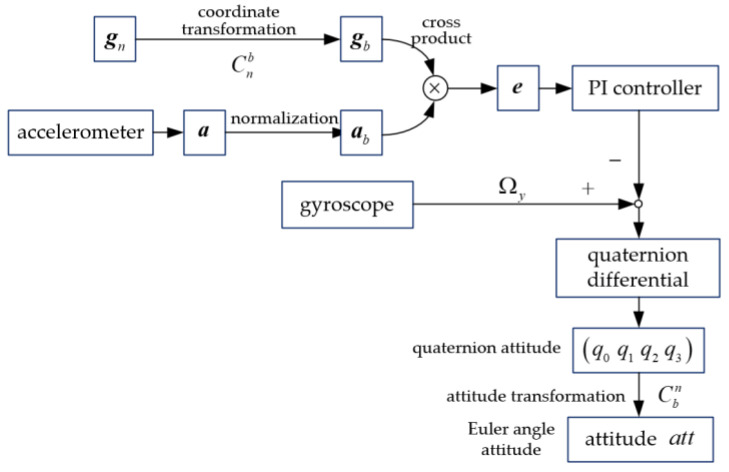
Mahony complementary filtering algorithm.

The Mahony complementary filtering algorithm is shown in Figure 5.

## 4. Experimental Simulations

In order to compare the attitude estimation results of Mahony complementary filtering and extended Kalman filter algorithms, and validate the feasibility of Mahony on the hardware platform, this paper builds a hardware platform based on STM32F107 and puts the flight control hardware (Holybro Pixhawk 4, Shenzhen Heli Brothers Technology Co., Ltd., Shenzhen, China) on a portable mobile robot as a controller. After transferring the signals to the controller, the data are transferred to the host computer through computer COM ports.

**Figure 6:**
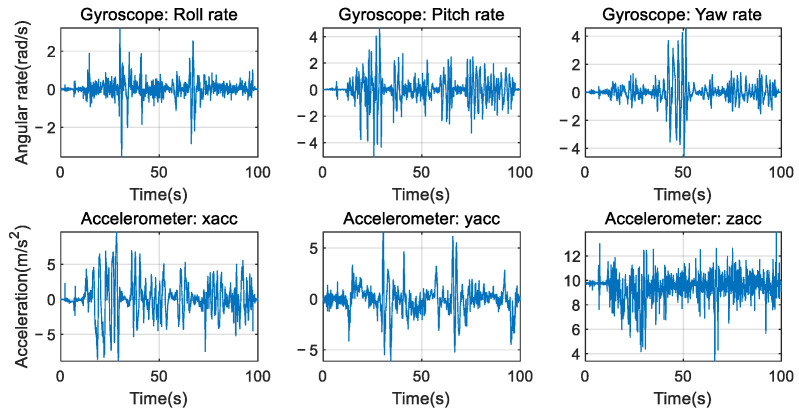
Data from gyroscope and accelerometer.

Figure 6 shows the signals from the gyroscopes and accelerometers, which are collected by the inertial measurement unit from Holybro Pixhawk 4 when the robot is in manual control motion conditions, and we upload the data into PC to do further analysis. The sampling frequency is 50 Hz.

**Figure 7:**
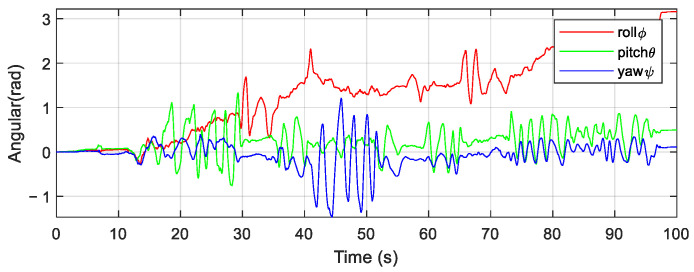
Attitude estimation by integrating gyroscope data.

**Figure 8:**
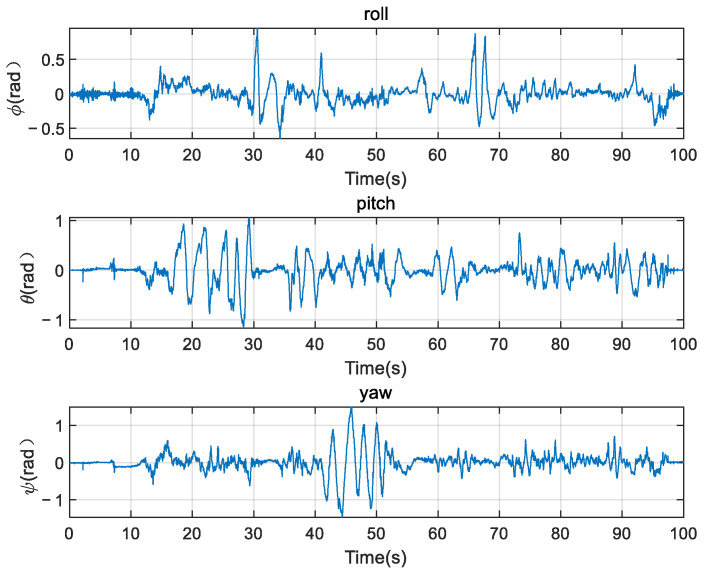
Attitude estimation by cooperating with accelerometers and magnetometers.

It can be seen from the three-axis attitude data in Figure 6 that both the gyroscope and the accelerometer have greater interference whenever in the motion or static state. If sensors’ data are not filtered, integrating the gyroscope directly to get the attitude of the robot according to Equation (11) will result in big errors caused by low-frequency noise. As is shown in Figure 7, especially the roll angle ϕ diverges. If we obtain the attitude angles by orthogonally decomposing the acceleration according to Equation (12), there will appear to be high-frequency noise, which will result in the angles’ inaccuracy, as is shown in Figure 8.

**Figure 9:**
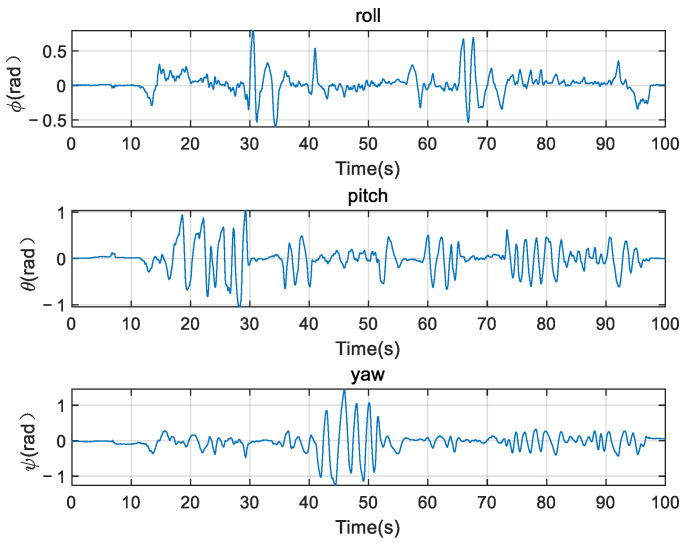
Attitude estimated by the PixHawk flight control hardware based on PX4-CF.

To better analyze the filtering results, take the robot attitude data estimated by the PixHawk flight control hardware into consideration. As is shown in Figure 9, the attitude data are estimated by the open-source PX4 flight stack based on the sensor measurements using the PX4 complementary filtering algorithm (PX4-CF) [26].

Based on the above simulation and analysis, the quaternion extended Kalman filtering (EKF) in Reference [5], linear complementary filtering (CCF) in Reference [21], and Mahony complementary filtering algorithms are utilized to fuse the data. In a linear complementary filtering algorithm, the cut-off frequency is 2 Hz after analyzing the frequency domain characteristics of the sensors’ signal. The proportional gain is 1 and the integral gain is 2 in the Mahony complementary filtering algorithm. To better analyze the filtering results, treat the average value of the estimated attitude angles based on PX4-CF, quaternion EKF, CCF, and Mahony as the ground truth.

**Figure 10:**
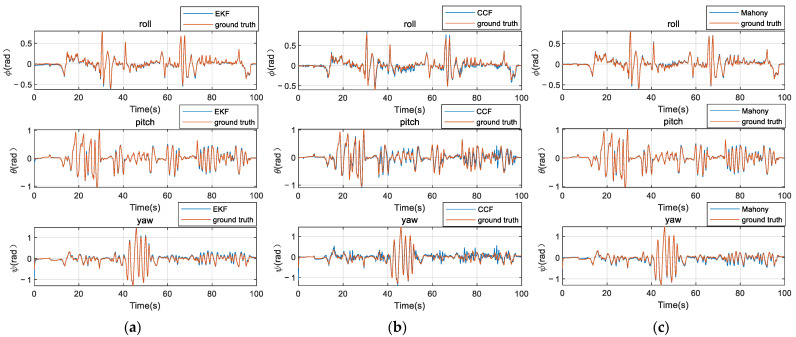
Attitude estimation: (**a**) quaternion extended Kalman filtering (EKF); (**b**) linear complementary filtering (CCF); (**c**) Mahony complementary filtering (Mahony).

The robot attitude estimation results are shown in Figure 10.

It can be seen from Figure 10 that the attitude angle can be effectively estimated by using quaternion EKF, CCF, and Mahony complementary filtering algorithms. When quaternion EKF is implemented, due to the randomness of the initial value in the algorithm, the estimated value of the attitude angles is quite different from the true value at the beginning. However, as iterations increase, the estimated value of the attitude angle gradually converges to the true value, which has a fast convergence rate. The results of CCF are less impressive, but it has a simple calculation with fast response. Mahony complementary filtering inherits the advantages of EKF, the convergence rate is fast, and the estimated attitude angles quickly converge to the true value at the initial state.

To compare the filtering algorithms in detail, we calculate the mean absolute error (MAE) of the attitude angle that the four algorithms estimated according to Formula (22). We treat the average values of the estimated attitude angles based on these four algorithms (PX4-CF, quaternion EKF, CCF, and Mahony) as ground truth. At the same time, the three algorithms (quaternion EKF, CCF, and Mahony) are implemented on the PC Intel(R) Core(TM) i5-10500 CPU (3.10 GHz) to calculate the average time consumption of the attitude estimation at each step. The results are shown in Table 1.

(22) $$MAE=\frac{1}{m}\sum_{i=1}^{m}\left|{\hat{y}}_{i}-y_{i}\right|$$

According to the data shown in Table 1, when it comes to the absolute mean error of PX4 complementary filtering, quaternion extended Kalman filtering, and Mahony complementary filtering algorithm, there is little difference between these three algorithms. In terms of the time consumption during attitude estimation, the quaternion EKF is nearly 10 times as long as the Mahony complementary filter algorithm. The results show that the Mahony complementary filtering algorithm has less computational cost than the quaternion extended Kalman filtering algorithm when the attitude estimation accuracy of these two algorithms is similar, which indicates that Mahony can be better applied to low-cost embedded systems. At the same time, when comparing classic linear complementary filtering and Mahony complementary filter algorithm, the computational cost of these two is similar, but the mean absolute error of Mahony’s complementary filtering algorithm is smaller than that of the linear complementary filtering algorithm.

## 5. Conclusions

To deal with the problem of high computational cost when using an extended Kalman filter (EKF) and particle filter (PF), this paper applies the complementary filtering algorithms in a low-cost portable mobile robot, which enables the low-cost embedded system to reduce the time consumption without reducing the accuracy of attitude estimation. Simulation experiments are carried out on the quaternion extended Kalman filtering algorithm, linear complementary filtering algorithm, and Mahony complementary filtering algorithm. Some concluding remarks are drawn as below:

(1) Based on the STM32F107 hardware platform, integrating the data from the gyroscope directly to get the attitude of the robot will result in big errors and even the estimated angles will diverge owing to the low-frequency noise. If the attitude angles are obtained by orthogonally decomposing the acceleration, high-frequency noise will appear, which will result in angles inaccuracy.
(2) Based on the simulation experiments when using the quaternion extended Kalman filtering algorithm, due to the randomness of the initial value in the algorithm, the estimated value of the attitude angles is quite different from the true value at the beginning. However, as the iterations increase, the estimated value of the attitude angles gradually converge to the true value, with a fast convergence rate.
(3) Based on the simulation experiments when using the complementary filtering algorithm, CCF is less impressive, but it has a simple calculation with fast response. Mahony complementary filtering inherits the advantages of EKF, the convergence rate is fast, and the estimated attitude angles quickly converge to the true value at initial.
(4) For the absolute mean error, there is little difference between quaternion extended Kalman filtering and the Mahony complementary filtering algorithm, but the absolute mean error of the linear complementary algorithm is bigger than that of these two algorithms.
(5) In terms of the time consumption during attitude estimation, there is little difference between the linear and Mahony complementary filtering algorithms. However, the Mahony complementary filter algorithm is nearly 10 times as fast as the quaternion Kalman filter.

According to the above discussions and analysis, we can see that the Mahony complementary filtering algorithm has less computation cost than the extended Kalman filtering algorithm when the attitude estimation accuracy of this two algorithms is similar, and it is suitable for low-cost embedded systems.

## Figures and Tables

**Table 1:** Mean absolute error of attitude angles and computational cost under different algorithms.

Algorithm	PX4-CF [26]	Quaternion EKF [5]	CCF [21]	Mahony
roll angle *φ*(*rad*)	0.0125	0.0136	0.0252	0.0129
pitch angle *θ*(*rad*)	0.0218	0.0213	0.0612	0.0215
yaw angle *ψ*(*rad*)	0.0269	0.0320	0.0737	0.0387
average time (*s*)	—	3.56 × 10^−5^	4.46 × 10^−6^	3.49 × 10^−6^

## References

[1] Peng X.D., Zhang T.M., Li J.Y. (2015). Attitude estimation algorithm of agricultural small-UAV based on sensors fusion and calibration. J. Acta Autom. Sin..

[2] Li B., Pan H.H., Chen L., Mao H.L. (2015). A data fusion algorithm for improving the detection precision of the robot end attitude. J. Mach. Des. Manuf..

[3] Qin Y.Y. (2006). Inertial Navigation.

[4] Grewal M.S., Weill L.R., Andrews A.P. (2001). Global Positioning Systems. Inertial Navigation and Integration.

[5] Wang X.Y., Yan J.H., Qin Y. (2007). Attitude estimation based on extended Kalman filter for a two-wheeled robot. J. Harbin Inst. Technol..

[6] Lu Y.J., Chen Y.D., Li Y.L. (2019). Experimental Study on Attitude Algorithm of Quadrotor Aircraft. J. Electron. Opt. Control.

[7] Choukroun D., Bar-Itzhack I.Y., Oshman Y. (2006). Novel quaternion Kalman filter. J. Aerosp. Electron. Syst. IEEE Trans..

[8] Bao Y.Q., Chen G.G., Wu K., Wang X.R. (2008). Research on Attitude Determination Using Magnetometers and MEMS Inertial Sensors. J. Acta Armamentarii.

[9] Xu M.M., Bu X.Z., Yang H.Q. (2020). Dual-Band Infrared and Geomagnetic Fusion Attitude Estimation Algorithm Based on IMMEKF. J. IEEE Trans. Ind. Electron..

[10] Wang X.X., Fan Q., Huang H. (2012). Overview of deterministic sampling filtering algorithms for nonlinear system. J. Control Decis..

[11] Liang S., Xu X.S., Huang Y.L. (2012). Application of Sage-Husa Adaptive Filter to Integrated Navigation System. J. Test Meas. Technol..

[12] Li G., Zhao D.Y., Xie R.C., Han H.L., Zong C.F. (2015). Vehicle State Estimation Based on Improved Sage-Husa Adaptive Extended Kalman Filtering. J. Automot. Eng..

[13] Zhang X.J., Xu Z.H., Yang S.P. (2018). Approach to Robot Attitude Algorithm. J. Mach. Des. Manuf..

[14] Li T.C., Fan H.Q., Sun S.D. (2015). Particle filtering: Theory, approach, and application for multi-target tracking. J. Acta Autom. Sin..

[15] Liang Y.D., Cheng M., He F.B. (2011). Attitude estimation of a quad- rotor aircraft based on complementary filter. J. Transducer Microsyst. Technol..

[16] Zhang R.H., Jia H.G., Chen T. (2008). Attitude solution for strapdown inertial navigation system based on quaternion algorithm. J. Opt. Precis. Eng..

[17] Chen M.Y., Xie Y.J., Chen Y.D. (2015). Attitude estimation of MEMS based on improved quaternion complementary filter. J. Electron. Meas. Instrum..

[18] D K. (2014). Nexter rolls out new Nerva UGV. J. Jane’s Int. Def. Rev. IHS Jane’s Int. Def. Rev..

[19] Zhang S.Q., Zhao Y.W. (2007). Attitude Algorithm of Portable Mobile Robot. J. Microcomput. Inf..

[20] Zhao X., Du P.X., Li H., Yang H.M. (2012). Attitude Estimation System based on MEMS accelerometer and gyroscope. J. Railw. Comput. Appl..

[21] Jung D., Tsiotras P. (2007). Inertial Attitude and Position Reference System Development for a Small UAV. AIAA.

[22] Premerlani W., Bizard P. (2009). Direction Cosine Matrix IMU: Theory. Diy Drone USA.

[23] Mahony R., Hamel T., Pflimlin J.M. Complementary filter design on the special orthogonal group SO(3). Proceedings of the 44th IEEE Conference on Decision and Control.

[24] Mahony R., Hamel T., Pflimlin J.M. (2008). Nonlinear complementary filters on the special orthogonal group. J. IEEE Trans. Autom. Control.

[25] Meng W., Guan L., Gao Y., Xu X., Xiong D. UAV Attitude Measurement based on Enhanced Mahony Complementary Filter. Proceedings of the 2018 IEEE International Conference on Mechatronics and Automation (ICMA).

[26] Battiston A., Sharf I., Nahon M. (2019). Attitude estimation for collision recovery of a quadcopter unmanned aerial vehicle. J. Int. J. Robot. Res..

